# Regulatory roles of circular RNAs in poultry diseases: mechanisms and research advances

**DOI:** 10.3389/fvets.2025.1626464

**Published:** 2025-11-12

**Authors:** Sumayya, Hassan A. Rudayni, Muhammad Shuaib, Bichun Li, Hongyan Sun, Obaid Ullah, Abdullah S. Alawam, Ahmed A. Allam, Ahmed A. Elolimy, Mohamed E. Abd El-Hack

**Affiliations:** 1 Key Laboratory of Animal Breeding, Reproduction and Molecular Design for Jiangsu Province, College of Animal Sciences and Technology, Yangzhou University, Yangzhou, China; 2 Department of Biology, College of Science, Imam Mohammad Ibn Saud Islamic University (IMSIU), Riyadh, Saudi Arabia; 3 Department of Veterinary Clinical Sciences, Faculty of Veterinary and Animal Sciences, University of Poonch, Rawalakot, Pakistan; 4 Department of Integrative Agriculture, College of Agriculture and Veterinary Medicine, United Arab Emirates University, Abu Dhabi, United Arab Emirates; 5 Department of Industrial Pharmacy, College of Pharmaceutical Sciences and Drug Manufacturing, Misr University for Science and Technology (MUST), Giza, Egypt

**Keywords:** circular RNAs, biogenesis, virus, regulatory mechanism, poultry diseases

## Abstract

Research on circular RNA (circRNA) in poultry increasingly explores its role as a potential regulator in various diseases. It has also shown differential expression of circRNAs in infected tissues, signifying their participation in disease pathogenesis and immune response mechanisms. Additionally, it can function as microRNA sponges, blocking the binding of microRNAs to their target mRNAs and modifying the expression of specific genes. circRNAs are being explored as potential biomarkers for early disease detection and monitoring of disease progression. They are highly expressed and stable in various chicken tissues, offering new insights into their role in tumor development. circRNAs are also being researched as a tool for gene editing, where they could potentially be utilized to increase or repress particular genes in therapeutic contexts. The vast quantity of circRNAs with unknown functions in the eukaryotic transcriptome means that, despite recent progress, our knowledge of the crucial role of circRNAs in viral infections and antiviral immune responses is still unclear. circRNA is a viable contender as a biomarker for bacterial infections due to its stability and distinct expression patterns. Numerous viral disorders have been found to exhibit altered circRNA expression profiles, suggesting their potential use in disease diagnostics. This review briefly introduces the biogenesis, characteristics, and functions of circRNAs, focusing on their progress in research on poultry diseases.

## 1 Introduction

Circular RNA (circRNA) is a non-coding, single-stranded RNA formed by back-splicing of precursor mRNAs (pre-mRNAs), a process where a downstream splice donor site is joined to an upstream splice acceptor site, creating a covalently closed circular structure. Research on circRNA in poultry is increasingly exploring its role as a potential regulator in various avian diseases, particularly in the context of viral infections like Marek’s disease virus (MDV) and avian leukosis virus (ALV), where several studies demonstrate differential expressions of circRNAs in infected tissues, suggesting their involvement in disease pathogenesis and immune response mechanisms. High-throughput RNA sequencing now profiles circRNAs in chicken tissues, identifying novel disease-associated species (1). circRNA structure is more stable and conserved than linear RNA, as circRNA forms a covalently closed loop without a 5′ end cap or a 3′ poly(A) tail (2). circRNAs typically range from 1 to 5 exons, with about 25% retaining introns in their mature form (3). Recent findings suggest the half-life of circRNAs varies between 8 and 50 h, and their peculiar circular shape allows them to be resistant to RNA exonucleases, resulting in stable expression (1). circRNAs are found either in the nucleus, especially multiexon-circles with retained introns, or in the cytoplasm, where most circRNAs primarily reside (4). Due to their covalently closed structure and resistance to exonucleases, circRNAs exhibit tissue-specific expression and are stable, making them promising diagnostic biomarkers. Moreover, circRNAs have been identified as potential treatment goals for various diseases, such as diabetes, heart disease, neurological conditions, chronic inflammation, and cancer. Their enrichment in exosomes (small extracellular vesicles) further supports their potential as diagnostic markers.

circRNAs have been detected in eukaryotes, including fungi, protists, plants, worms, fish, insects, and mammals, exhibiting cell-type- and tissue-specific expression patterns, often conserved across species and developmental stages (5). These molecules act as microRNA sponges, up-regulating target mRNA expression, and are involved in multiple regulatory functions, such as transcriptional and translational control, protein interaction facilitation, and even direct translation into proteins (6). For instance, circFOXO3 interacts with proteins associated with stress and senescence, inhibiting their nuclear translocation and mitigating senescence and stress in cardiac fibroblasts (7).

As biotechnology and molecular medicine have developed, artificial circRNAs have been created as innovative vaccines for both the prevention and therapy of disease (8). Since circRNAs can show proteins correctly in cells and exhibit reduced immunogenicity, synthetic purification circRNAs are appropriate for *in vivo* applications, like vaccines or therapeutic agents. circRNA vaccines and gene therapies have gained importance, and further research into circRNAs has highlighted their crucial functions in both physiological and pathological mechanisms, particularly in regulating gene expression at multiple levels (9).

In poultry, certain circRNAs, including circRNA_3238 and circRNA_3079, have been implicated in avian leukosis virus (ALV) infection (10). circRNAs are highly expressed and stable in various chicken tissues, offering new insights into their role in tumor development in poultry (11). Qiu et al. (12) explored circRNA expression in both healthy and ALV-J-infected chicken spleen tissues, indicating a role for circRNAs in tumor genesis. Researchers are investigating the specific functions of identified circRNAs by studying their interaction with miRNAs and the downstream effects on gene expression (3).

Given their ubiquitous presence and diversity, circRNAs likely play significant roles in both normal cellular physiology and pathological processes. Further research is required to elucidate the detailed molecular process by which circRNAs control gene expression and contribute to disease pathogenesis. We hypothesize that circRNAs function as key regulatory molecules that modulate disease pathogenesis and immune responses in poultry through multiple mechanisms, including miRNA sponging, protein scaffolding, and transcriptional regulation. This review aims to test this hypothesis by examining the current evidence for circRNA involvement in poultry diseases and their potential as diagnostic biomarkers and therapeutic targets. Therefore, this review briefly introduces the properties, roles, and biogenesis of circRNAs, emphasizing their research progress in poultry diseases. Specifically, Section 2 provides historical context; Sections 3 and 4 describe circRNA biogenesis and characteristics; Section 5 focuses on regulatory mechanisms; Sections 6 and 7 review circRNAs in poultry diseases; and Section 9 concludes with future perspectives.

## 2 Historical background

Understanding the historical progression of circRNA research provides crucial context for evaluating our hypothesis that circRNAs function as key regulatory molecules in poultry diseases, as the evolution from “splicing artifacts” to recognized regulatory elements mirrors their potential importance in avian pathology. Circular RNAs were first identified in 1976 when Sanger et al. observed RNA circles in viroids using electron microscopy (13). Soon after, Hsu and Coca-Prados detected them in bodily fluids, including the cytoplasm of human HeLa cells, and in 1980 they were also found in the mitochondrial genome of yeast (14). Initially thought to be exclusive to viroids, circRNAs were later detected in the hepatitis delta virus in 1986 (15), and in adenoviruses infecting animal cells. At the time, they were considered rare and biologically insignificant splicing by-products in mammals, although early evidence hinted at their presence in higher organisms (16). In the 1990s and early 2000s, genes producing circRNAs were found across eukaryotes from flies to mammals, but many discoveries were dismissed during the so-called “Artifact Era” due to detection limitations and the challenge of distinguishing them from linear RNAs (17). By the mid-2000s, advances in bioinformatics and high-throughput sequencing allowed reliable identification of circRNAs as distinct RNA species, prompting recognition of their potential regulatory roles in gene expression (18).

**Figure 1:**
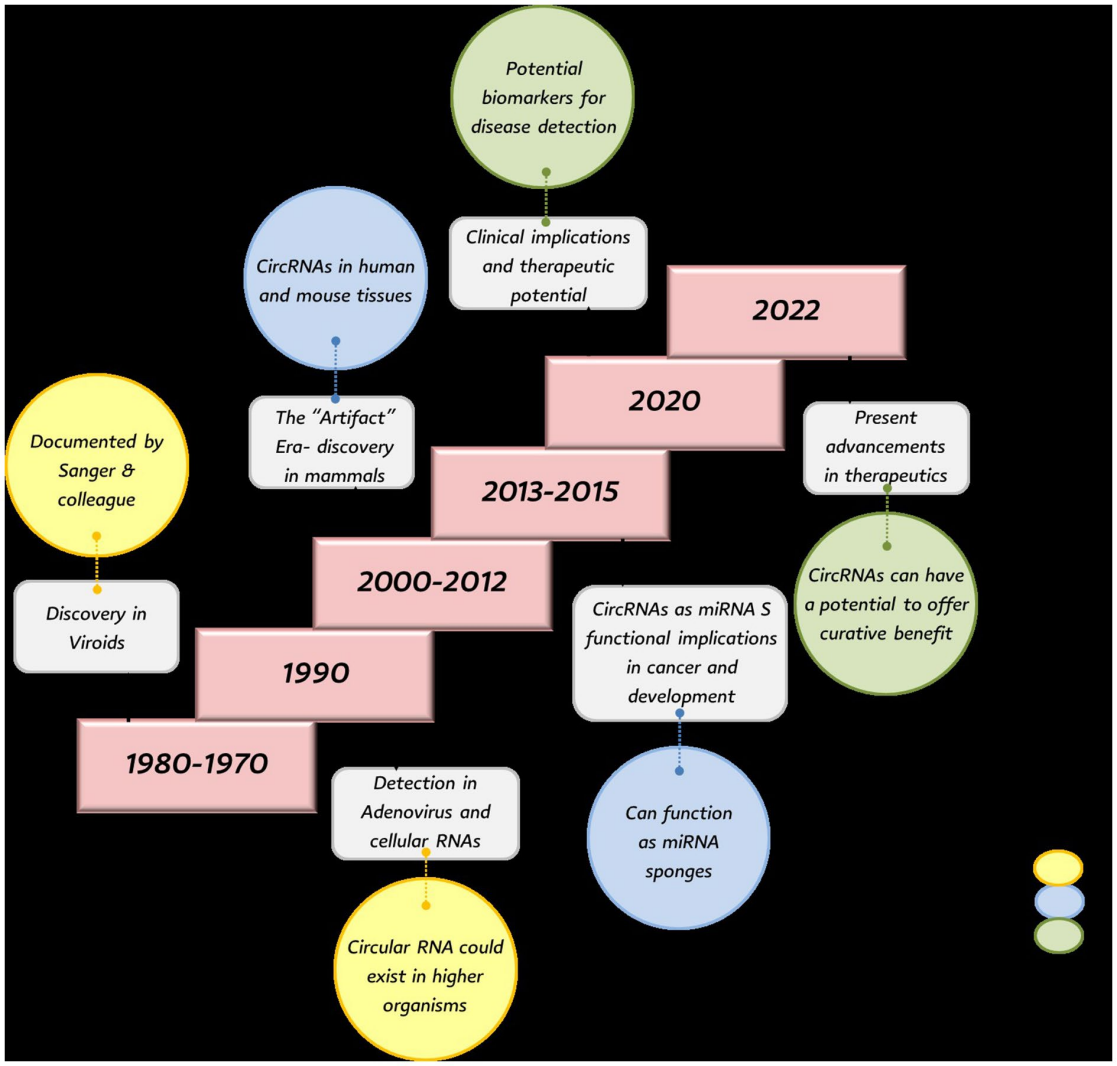
Historical background of circular RNAs.

In 2012, high-throughput RNA sequencing (RNA-seq) transformed non-coding RNA research and revealed numerous circRNAs in mammalian cells. A landmark study by Salzman et al. (18) showed that circRNAs are abundant, stable, and not splicing errors, but rather a distinct class of transcripts with potential biological functions. This marked a paradigm shift in RNA biology, distinguishing circRNAs from linear mRNAs. Subsequent studies revealed that circRNAs can serve as crucial regulators of cellular processes. Advances in RNA-seq have since confirmed that thousands of circRNA types are expressed across metazoans. Figure 1 summarizes the historical background of circRNAs.

Following their discovery, circRNAs were recognized for important regulatory functions. Hansen (19) demonstrated that they can act as “miRNA sponges,” sequestering microRNAs (miRNAs) to prevent them from binding target mRNAs, thereby influencing gene expression. Jeck (20) linked circRNAs to cell growth, differentiation, and apoptosis; for instance, CDR1as (ciRS-7) regulates cancer cell proliferation by binding miR-7, a microRNA involved in oncogenic pathways. circRNAs are now associated with cancer, neurological disorders, and cardiovascular diseases, with their stability and detection in blood and saliva making them promising biomarkers (21). In cardiovascular contexts, they modulate key pathways critical for heart function (22). Increasingly, their therapeutic potential is being explored through approaches such as CRISPR-Cas9 and RNA interference to modulate expression for treating cancers, neurological conditions, and other diseases (23). Once considered splicing by-products, circRNAs are now established as multifunctional regulators in diverse cellular processes, including immune responses and pathogen-related pathways. Their stability, tissue-specific patterns, and presence in biofluids position them as strong candidates for diagnostic and therapeutic applications.

Although early circRNA research focused on mammals, poultry studies have expanded rapidly due to the economic impact of infectious diseases and interest in host–pathogen interactions. The first avian-focused work in the late 2010s profiled circRNA expression across tissues, with (24) linking circRNAs to resistance against avian leukosis virus subgroup J (ALV-J)-induced tumors, the first evidence that host circRNAs modulate oncogenic viral outcomes in any species. Subsequent studies implicated circRNAs in responses to Newcastle disease virus (NDV) and *Eimeria tenella*, connecting them to immune pathways such as NF-κB and B cell receptor signaling (25, 26). Chen et al. (25) uncovered 86 NDV-responsive circRNAs in chicken embryo fibroblasts and showed that over-expression of circ-EZH2 inhibited velogenic NDV replication. Most recently, synthetic circRNAs engineered for low immunogenicity have entered pre-clinical pipelines as next-generation poultry vaccines (8).

In Marek’s disease (MD), circRUNX2.2 promoted lymphoma cell proliferation via RUNX2 activation, indicating an oncogenic role (27), and genome-wide profiling revealed both host- and virus-derived circRNAs. ALV-J studies reported differential expression in resistant birds (12) and a ~30% circRNA loss in infected ones, with circHRH4 showing stability and possible tumorigenic involvement (28, 29). In *E. tenella* infection, specific circRNAs were linked to adaptive immunity and NK cell activity (26). circRNA changes have also been observed under ammonia exposure and in infectious bursal disease (30).

Beyond disease, circRNAs influence productivity traits. In muscle, circTMTC1, circFGFR2, circHIPK3, and circSVIL regulate myoblast proliferation and differentiation via miRNA interactions (30, 31). In abdominal fat, circRNAs associated with lipid metabolism and mTOR/TGF-β pathways provided the first comprehensive fat-tissue profiles (32, 33). Overall, poultry circRNAs are emerging as key regulators in disease, immunity, and production traits, with diagnostic and therapeutic potential. Collectively, this historical trajectory demonstrates how circRNAs have evolved from overlooked splicing byproducts to recognized regulatory molecules across species, providing strong foundational evidence supporting our hypothesis that they serve as key modulators of disease pathogenesis and immune responses in poultry through multiple regulatory mechanisms.

## 3 circRNA biogenesis and classification

Elucidating the biogenesis and classification of circRNAs is fundamental to testing our hypothesis, as understanding how these molecules are generated and categorized reveals the mechanistic basis for their regulatory potential in poultry disease contexts. The first phase of circRNA synthesis involves the transcription of eukaryotic precursor mRNAs (pre-mRNAs) by RNA polymerase II, followed by splicing to excise introns and ligate exons, resulting in mature mRNAs (34). circRNA is produced via a unique kind of alternative splicing called head-to-tail splicing or back-splicing, in which an exon’s 3′ end ligates to the 5′ end of either its own or an upstream exon via 3′–5′ phosphodiester bonds. This produces a closed-loop configuration, including a back-splicing junction point. Initially, circRNAs were considered splicing anomalies, including “scrambled exons” (35).

circRNAs are classified into three types: exonic, exon-intron, and circular intronic based on their composition: exonic circRNAs (ecircRNAs) composed only of exons, exon-intron circRNAs (EIciRNAs) including both exons and introns, and circular intronic RNAs (ciRNAs) originating from introns (36). More than 80% of known circRNAs are ecircRNAs, mostly located in the cytoplasm. Conversely, EIciRNAs and ciRNAs are primarily situated in the nucleus, where they are believed to be involved in the control of gene transcription (37). Circular RNAs can modulate gene transcription in the nucleus by interacting directly with the promoter region of their parental genes, recruiting transcription factors or RNA polymerase II, and forming RNA–DNA hybrid structures (R-loops). Depending on the specific circRNA and cellular context, this can either enhance or repress transcription, functioning as a “scaffold” for the transcriptional machinery at the gene locus. The three canonical circularization models (intron-pairing, lariat-driven, RBP-assisted) were established in mammalian cells; orthologous intronic architecture and repeat elements have been reported in chickens (e.g., reverse-complementary matches overlapping CR1 repeats), supporting—but not proving—mechanistic conservation in avian species.

Recent studies have identified three models of biosynthesis of circRNA that reflect the contest between back-splicing and canonical splicing: (i) Intron-pairing-driven circularization, (ii) lariat-driven circularization or exon skipping, and (iii) RNA-binding protein (RBP)-driven circularization (38). In the intron-pairing concept, back-splicing is promoted by base-pairing among different introns, especially involving repetitive sequences such as ALU repeat. In the lariat-driven model, circularization occurs when exons are eliminated for creating linear RNA, allowing splice site joining. In the RBP-driven model, Introns close to splice sites are bound by RBPs, promoting the formation of circular RNAs (39). Translation, transcription or splicing regulation, protein interaction, and RNA interaction represent a few of the possible processes of circRNAs. Circular RNAs can also be released into extracellular space after being enclosed in exosomes, which are produced by vesicle cells.

Circular RNAs arise from spliceosome-mediated back-splicing of pre-mRNAs through three routes: intron pairing (complementary repeats bring flanking splice sites together), lariat-driven back-splicing (exon skipping followed by processing of the intron lariat), and RNA-binding protein–assisted circularization (RBPs bridge flanking introns). The figure also summarizes key functions: regulate transcription/splicing, sponge miRNAs and interact with mRNA/lncRNA (ceRNA), bind proteins as decoys/scaffolds, sort into exosomes for extracellular release, and translate into peptides via cap-independent initiation (IRES/m^6^A). Together, these biogenesis mechanisms and classification systems illustrate how circRNAs are generated through highly regulated processes that determine their cellular localization and functional capacity, supporting our hypothesis that their diverse origins and structural features enable them to function as versatile regulatory molecules in poultry disease pathogenesis and immune responses.

## 4 Characteristics of circRNAs

The unique structural and functional characteristics of circRNAs provide the molecular foundation for testing our hypothesis, as their stability, tissue specificity, and conservation across species suggest they are well-positioned to serve as key regulatory molecules in poultry health and disease. Circular RNAs are a unique class of single-stranded, covalently closed RNA molecules found across a wide range of species, including eukaryotes such as animals and plants. They are primarily generated from precursor messenger RNAs (pre-mRNAs) through a non-canonical process called back-splicing, in which a downstream splice donor site is joined to an upstream splice acceptor site. Unlike linear RNAs, circRNAs lack both a 5′ cap and a 3′ poly(A) tail, a structural feature that makes them highly resistant to exonuclease-mediated degradation, such as by RNase R, and results in remarkable stability, often two to five times greater than that of their linear counterparts (40, 41). This closed-loop structure, combined with their widespread occurrence, enhances their potential as biomarkers and therapeutic targets in clinical and veterinary research (42–44). General features such as exon dominance, nuclear versus cytoplasmic partitioning, and RNase R resistance derive primarily from mammalian studies; where chicken-specific distributions or exceptions have been experimentally validated (e.g., tissue-specific expression hotspots, developmental stage specificity in Gushi chickens), they are explicitly noted with avian data references.

In poultry, circRNAs are broadly distributed across all chromosomes, including sex chromosomes, with certain genomic regions acting as hotspots for circularization events. They vary greatly in length, from under 100 nucleotides to nearly 100,000 nucleotides, depending on the tissue and breed. Most identified chicken circRNAs are derived from exonic regions, 84.95% in Gushi chicken adipose tissue and 81.5% in pectoralis muscle, while a smaller proportion originates from introns, intergenic sequences, or untranslated regions (45). The expression of circRNAs is often tissue-specific and developmentally regulated, with some showing distinct patterns such as bimodal expression peaks in Gushi chicken adipose tissue (45, 46). Their expression is generally lower than that of linear mRNAs, yet their stability allows them to accumulate and persist in specific cell types. External factors, such as ammonia exposure, viral infections (including Avian Leukosis Virus subgroup J, Marek’s disease virus, and H5N1 avian influenza virus), bacterial infections (*Salmonella enterica* serovar Enteritidis), and even insulin administration, can dynamically regulate circRNA expression in chickens (47).

Functionally, circRNAs participate in a diverse range of biological processes. Many act as microRNA (miRNA) sponges, sequestering specific miRNAs and thus modulating post-transcriptional gene regulation (42, 44). Others interact with RNA-binding proteins (RBPs), influence transcription, affect pre-mRNA splicing, and, in some cases, may even serve as templates for protein or peptide translation, particularly when they contain internal ribosome entry site (IRES) elements. While translation has been experimentally confirmed for some mammalian circRNAs, definitive evidence in poultry or plants remains lacking, though the potential is strong. Subcellular localization also correlates with function, exonic circRNAs are predominantly cytoplasmic, whereas intronic and exon–intron circRNAs are often nuclear (48).

Circular RNAs are highly conserved across species, indicating their evolutionary and functional importance. For example, circINSR, derived from the insulin receptor (INSR) gene in chickens, shares approximately 77% sequence similarity with its mammalian counterparts, including humans, mice, and bovines (47). This conservation supports the idea that circRNAs carry out essential, preserved roles in cellular regulation. Their stability, tissue specificity, and involvement in key physiological and pathological processes, including muscle development, fat metabolism, and immune responses, underscore their potential not only as diagnostic and prognostic biomarkers but also as targets for therapeutic interventions (29, 46). Given their multifaceted biological significance, continued in-depth studies are essential to fully understand their functions and translate these findings into practical applications for both human and animal health (43, 49, 50). Collectively, these distinctive characteristics, including exceptional stability, tissue-specific expression, evolutionary conservation, and environmental responsiveness, provide compelling evidence supporting our hypothesis that circRNAs function as key regulatory molecules in poultry, with their unique properties enabling them to effectively modulate disease pathogenesis, immune responses, and serve as reliable diagnostic biomarkers and therapeutic targets.

## 5 Regulatory mechanism of circRNA

The diverse regulatory mechanisms employed by circRNAs provide direct evidence for testing our central hypothesis, as their ability to function through multiple pathways—including miRNA sponging, protein scaffolding, translation, and transcriptional regulation—demonstrates their capacity to serve as key modulators of disease pathogenesis and immune responses in poultry. Circular RNAs serve multiple functions within the cell. One of their most extensively studied roles is as master regulators of gene expression. They can act as competing endogenous RNAs (ceRNAs), sequestering or “sponging” regulatory molecules, particularly microRNAs (miRNAs) (51). Additionally, some circRNAs have the capacity to be transformed into proteins. Since circRNAs can encode proteins in cells while eliciting minimal immunogenicity, synthetic circRNAs are promising for *in vivo* applications such as vaccines or therapeutic agents. Mechanistic models summarized below are largely defined in mammalian systems; poultry-specific validations are called out in each subsection. Table 1 summarizes only avian-derived circRNA mechanisms and phenotypes (species/cell type, disease/process, outcomes, targets).

**Table 1:** Poultry circRNA regulatory mechanisms, targets, and phenotypes (avian experimental evidence).

Regulatory mechanism/function	Specific circRNA (if named)	Poultry species/cell type	Disease/process	Key effect/outcome	Target molecules (if specified)	References
miRNA Sponge/ceRNA Network	circRUNX2.2 (gga_circ_0009437)	Chicken (tumorous spleens, MDCC-MSB1 cell line)	Marek’s disease (MD) lymphoma progression	Promotes proliferation of lymphoma cells; positively regulates RUNX2 expression	RUNX2 promoter region (cis-acting); recruits CHD9 protein (facilitates positive regulation of RUNX2)	(27)
	circZMYM3 (gga_circ_0011261)	Chicken (spleens)	Marek’s disease (MD) tumorigenesis	Involved in immune regulation and tumorigenesis	Seven miRNAs (e.g., gga-miR-155, which targets GATA4), immune genes (e.g., SWAP70, CCL4)	(56)
	circGTDC1, circMYO1B	Chicken (spleens)	Marek’s disease (MD) tumorigenesis	Involved in immune responses and tumorigenesis	Gga-miR-155, immune-related genes (e.g., GATA4)	(56)
	circRNA_3238, circRNA_3079	Chicken	Avian Leukosis Virus (ALV) infection, tumor genesis	Implicated in ALV infection and tumor development; circRNA_3079 may indirectly regulate ALV-J infection and is associated with immunity/tumors (p53, Jak–STAT pathways)	Unspecified miRNAs/target genes for circRNA_3238; p53 signaling pathway, Jak–STAT signaling pathway (for circRNA_3079)	(57)
	circHRH4 (circRNA_1193)	Chicken (spleen, various tissues)	Avian Leukosis Virus J (ALV-J) induced tumorigenesis	Particularly abundant and involved in tumorigenesis	Mll and Aoc3 (genes), cooperating with unspecified miRNAs	(28)
	circRBFOX2s	Xinghua Chicken (embryonic leg muscle)	Skeletal muscle development and differentiation	Promotes cell proliferation; can downregulate RBFOX-splicing factors	miR-206 and miR-1a (MREs)	(30, 58)
	circSVIL	Chicken (skeletal muscle)	Myogenesis, embryonic skeletal muscle development	Promotes myoblast proliferation and differentiation; enhances mRNA levels of MEF2C and c-JUN	miR-203	(30, 58)
	circTMTC1	Chicken (skeletal muscle satellite cells)	Myogenesis, skeletal muscle satellite cell differentiation	Inhibits differentiation	miR-128-3p	(30, 61)
	circFGFR2	Chicken (myoblasts, skeletal muscle)	Myoblast proliferation and differentiation	Promotes myoblast proliferation and differentiation	miR-133a-5p and miR-29b-1-5p	(58)
	circHIPK3	Chicken (myoblast cells)	Myoblast proliferation and differentiation	Promotes proliferation and differentiation	miR-30a-3p	(60)
	Z:54674624|54,755,962	Gushi Chicken (abdominal adipocytes)	Abdominal adipogenic differentiation	Functions as a ceRNA to regulate adipogenesis	gga-miR-1635 (targets AHR2, IRF1, MGAT3, ABCA1, AADAC) and/or novel_miR_232 (targets STAT5A)	(33)
	gga_circ_0002520	Gushi Chicken (abdominal adipose tissue)	Lipid metabolism, abdominal fat development	Plays critical role in fat development	miR-215-5p (targets NCOA3 mRNA)	(45)
	circRNA225, circRNA226	Chicken (breast muscle)	Muscle development (aging)	Contributes to glycolysis/gluconeogenesis, amino acid biosynthesis, pyruvate metabolism, carbon metabolism, glycogen/sucrose metabolism; affects postnatal muscle development by regulating muscle protein deposition	Seven common miRNAs (e.g., gga-miR-1306-5p), 207 mRNAs (e.g., HSPA8)	(59)
	circINSR	Arbor Acres (AA) broilers, Silky fowls (pectoralis muscle)	Insulin signaling, glucose homeostasis, muscle development	Early insulin responder; downregulated by insulin (in broilers at 15 min), energy restriction	Unspecified miRNAs (e.g., miR-103, miR-107, miR-143, miR-145, miR-26a)	(47)
	circRNA2202 and circRNA0759, circRNA6300, circRNA4338, circRNA2612	Chicken	*Eimeria tenella* infection	Involved in adaptive immune response, NF-kappa B signaling, natural killer cell-mediated cytotoxicity, B cell receptor signaling pathways	DTX1, *RORC* and *CD101,* VPREB3, CXCL13L3, IL8L1, F2RL2 (related mRNAs/genes)	(26)
	gga_circ_0004993, gga_circ_0003686, gga_circ_0001479, gga_circ_0009799, gga_circ_0006492, gga_circ_0000808	Chicken (Gushi breast muscle)	Skeletal muscle development and cell growth	Mediates skeletal muscle development through complex ceRNA networks	miR-206, miR-148a-3p, miR-499-5p, gga-miR-181b-5p (and other miRNAs); CCNT2, ACTA1, FOXP1, ANKRD1 (source genes)	(31)
	Unspecified	Chicken (abdominal adipose tissue)	Abdominal fat development, lipid metabolism	Regulate lipid metabolism, adipocyte proliferation and differentiation, and cell junctions	Unspecified miRNAs (e.g., let-7b, miR-101-3p, miR-103-3p, miR-10b-5p, gga-miR-33); pathways like propionic acid metabolism, fatty acid biosynthesis, pyruvate metabolism	(45)
ceRNA (miRNA sponge) controlling kinase/transcription factor nodes	10:12574340|12,607,185	Chicken abdominal preadipocytes	Adipogenesis	Interacts with gga-miR-92-5p and novel_miR_263 to influence lipid-regulatory signaling	STK10 (AMPK-related kinase axis), STAT5A	(33)
Regulation of Transcription	circRUNX2.2 (gga_circ_0009437)	Chicken (tumorous spleens)	Marek’s disease (MD) development	Promotes expression of its parental gene RUNX2 in a cis-acting manner	RUNX2 promoter region; recruits CHD9 protein	(27)
Protein/Peptide Translation	circZNF609	Human (myogenesis) (Mentioned as applicable to animals in general context)	Myogenesis, myoblast proliferation	Can be translated into a protein (driven by IRES); controls myoblast proliferation	Unspecified	(31)
	circFAM188B	Chicken (skeletal muscle)	Skeletal muscle development, proliferation	Encodes circFAM188B-103aa protein; promotes proliferation of chicken SMSC	Unspecified	(62)
RBP Interaction/Scaffolding	circRUNX2.2	Chicken (tumorous spleens)	Marek’s disease (MD) development	Can recruit proteins (e.g., CHD9 protein)	CHD9 protein	(27)
	circMbl	Animals (general context)	circRNA biogenesis, splicing factor regulation	Binding to MBL protein influences circMbl biosynthesis; can sponge out excess MBL protein to decrease its level	Muscleblind (MBL) protein binding sites in flanking introns	(48, 63)
Competition with Pre-mRNA Splicing	Z:35565770|35568133	Gushi Chicken (abdominal adipocytes)	Abdominal adipogenic differentiation	Might compete splicing with its parental gene ABHD17B	ABHD17B (parental gene)	(33)
Co-Expression Clustering Analysis of DE-circRNAs and DE-Genes	1:46678392| 46681235, 13:17449530|17452588, 2:102486533|102511558, 8: 27886180|27889657	chicken	adipogenesis	lipid metabolism	CPT1A, AADAC, ACSS2, ACSL1, AGPAT2, FABP4, CYP26B1, and FAAH	(33)
ceRNA (miRNA sponge) within fat-deposition networks	novel_circ_PTPN2, novel_circ_CTNNA1, novel_circ_PTPRD	Chicken (*Gallus gallus*); abdominal fat, back skin, liver	Fat deposition	circRNAs participate in ceRNA networks tied to PPAR and fatty-acid metabolic pathways; help regulate key lipid genes	miRNAs: gga-miR-460b-5p, gga-miR-199-5p, gga-miR-7470-3p, gga-miR-6595-5p, gga-miR-101-2-5p; genes: FADS2, HSD17B12, ELOVL5, AKR1E2, DGKQ, GPAM, PLIN2	(64)
ceRNA modules linked to PPAR/fatty-acid pathways (intramuscular vs. abdominal fat)	circLCLAT1, circFNDC3AL, circCLEC19A, circARMH1	Chicken adipocytes (intramuscular & abdominal)	Adipogenesis/tissue-specific fat deposition	Candidate circRNAs regulate miRNAs impacting PPAR and fatty-acid metabolism	(miRNAs not all named in abstracted section)	(65)
Global circRNA participation across tissues	Multiple DE circRNAs (41 in abdominal fat; 26 back skin; 15 liver)	Chicken; abdominal fat, back skin, liver	Fat deposition	Tissue-resolved ceRNA networks implicate lipid metabolism (PPAR, glycerolipid/FA processes)	Network involves circRNAs with miRNAs above and lipid genes listed	(64)

### 5.1 circRNAs regulate gene expressions as miRNA sponges

CircRNAs function as “super sponges” that provide quantitative control over miRNA availability, establishing them as master regulators that can amplify or dampen entire gene expression programs through single molecular interactions. This regulatory amplification model is supported by their superior miRNA-binding affinity compared to other endogenous RNAs and their capacity to sequester multiple miRNA copies simultaneously (52). The regulatory power of circRNA sponging is exemplified by CDR1as/CiRS-7, which demonstrates how a single circRNA can reshape entire cellular programs. With 73 binding sites for miR-7, CiRS-7 creates a molecular sink that depletes miR-7 availability throughout the cell, simultaneously derepressing all miR-7 target genes (53). This multiplexed regulation means that CiRS-7 expression changes do not simply adjust individual genes, they coordinately regulate entire miR-7-controlled genetic programs. The regulatory principal scales beyond individual circRNA-miRNA pairs to create hierarchical control networks. The 70 conserved miRNA target sites in circCDR1 illustrate how circRNAs can simultaneously regulate multiple miRNA families, creating regulatory hubs that integrate diverse cellular signals (54). This hub architecture explains why circRNA dysregulation has such profound pathological effects: disrupting a single circRNA node can cascade through multiple miRNA-controlled pathways, amplifying regulatory perturbations across entire cellular networks. In pathological contexts, this amplification transforms circRNAs from simple molecular sponges into disease-driving master regulators. Furthermore, circRNAs have been connected to many pathological processes through their ability to sponge different miRNAs. By competing with mRNAs for miRNA binding, circRNAs can negatively regulate miRNA activity, indirectly influencing mRNA levels. Consequently, circRNAs can alleviate or reduce the inhibition of genes targeted by miRNAs, thereby modulating the expression of those genes. For example, overexpression of circLRP6 has been shown to accelerate the sponging of miR-145 to lead to atherosclerosis (55).

Across poultry systems, circRNAs frequently act as competing endogenous RNAs (ceRNAs) that tune disease and development by sequestering miRNAs and derepressing key targets. In Marek’s disease (MD), *circRUNX2.2* (gga_circ_0009437) promotes lymphoma-cell proliferation by enhancing its parental gene in cis and recruiting CHD9 at the RUNX2 promoter, consistent with a ceRNA-coupled transcriptional mechanism (27). Additional MD-linked circRNAs—including *circZMYM3*, *circGTDC1*, and *circMYO1B*—are embedded in immune/tumorigenic ceRNA networks featuring gga-miR-155 and downstream immune genes such as GATA4, SWAP70, and CCL4 (56). In avian leukosis, *circRNA_3079* aligns with p53 and JAK/STAT signaling enrichment and likely modulates ALV-J infection via miRNA pathways, while *circHRH4* (circRNA_1193) is abundant across tissues during ALV-J tumorigenesis (28, 57). In skeletal muscle, *circRBFOX2s*, *circSVIL*, *circTMTC1*, *circFGFR2*, and *circHIPK3* govern myoblast proliferation/differentiation through ceRNA control of miR-206/miR-1a, miR-203, miR-128-3p, miR-133a-5p/miR-29b-1-5p, and miR-30a-3p, respectively (30, 58–60). Adipogenic ceRNA axes include *Z:54674624|54755962* (gga-miR-1635; novel_miR_232) and gga_circ_0002520 (miR-215-5p → NCOA3), with broader networks in breast muscle and abdominal fat engaging miR-206/148a-3p/499-5p/181b-5p and lipid-metabolism pathways (31, 45, 59). Notably, *circINSR* behaves as an early insulin responder in pectoralis muscle, linking ceRNA regulation to glucose and growth phenotypes (47). In coccidiosis, differentially expressed circRNAs associate with adaptive immunity, B-cell receptor signaling, NK-cell cytotoxicity, and NF-κB modules during *Eimeria tenella* infection, reinforcing an immune-centric ceRNA layer (26). These poultry-specific examples of circRNA-mediated ceRNA networks clearly demonstrate how circRNAs function as key regulatory molecules in avian systems, supporting our hypothesis through their documented roles in modulating viral pathogenesis, immune responses, muscle development, and metabolic processes via miRNA sequestration mechanisms.

### 5.2 Circular RNAs as protein decoys

circRNAs can directly engage with circRNA-binding proteins (cRBPs), serving as protein decoys that influence various cellular functions. By binding to specific proteins, circRNAs can control the translocation of these proteins within the cell. For instance, the circRNA circ-Ccnb1 forms a ternary complex with Ccnb1 and Cdk1, which inhibits the activity of Ccnb1, thereby promoting cell proliferation and survival (66). The interaction of circAmotl1 with PDK1 and AKT1 in primary cardiomyocytes results in the phosphorylation and nuclear translocation of AKT1, hence diminishing apoptosis and facilitating cardiac healing. Likewise, circFOXO3 promotes the interaction between MDM2 and p53, leading to reduced p53 protein levels and the consequent initiation of apoptosis (67).

CircACC1 is another example that contributes to metabolic adaptation to serum deprivation by increasing the enzymatic activity of the AMP-activated protein kinase (AMPK) holoenzyme. This transpires via establishing a ternary complex with the AMPK β and γ regulatory subunits (68). However, bioinformatics analyses indicate that circRNAs exhibit decreased RBP binding density compared to linear RNAs, suggesting that few circRNAs may be unable to effectively interact with proteins. Thus, there is a critical requirement for innovative approaches to facilitate more efficient studies on circRNA-protein interactions. Most protein-decoy/scaffold interactions (e.g., circFOXO3–MDM2–p53; circAmotl1–AKT1) are mammalian; direct RBP-binding demonstrations in poultry remain limited and are identified explicitly where available. Evidence for circRNA–RBP architecture in poultry includes circRUNX2.2 recruitment of CHD9 during MD progression (27); more broadly, animal models show that circMbl binds the splicing factor MBL to regulate its own biogenesis and buffer excess MBL—a mechanism likely conserved but still underexplored experimentally in avian tissues (48, 63). While most protein-scaffolding mechanisms derive from mammalian studies, the emerging evidence from poultry systems, particularly circRUNX2.2’s recruitment of CHD9, supports our hypothesis by demonstrating that avian circRNAs can indeed function as key regulatory molecules through direct protein interactions that influence disease progression.

### 5.3 circRNAs translation into proteins

Most circRNAs are found in the cytosol, suggesting their potential involvement in ribosome assembly for protein translation. Studies have demonstrated that circRNAs containing translation of an internal ribosome entry site (IRES) are available in peptides both *in vitro* and *in vivo* (67). While some circRNAs are localized in the nucleus, most are predominantly abundant in the cytoplasm. This cytoplasmic localization allows circRNAs to interact with proteins and microRNAs, thereby regulating gene expression (69). Recent research has shown that the translation of ecircRNAs, manufactured from back-splicing in human cells that have been vectorized, is indeed possible (70).

However, there is currently no evidence to suggest that spliceosome-generated ecircRNAs can function as mRNAs. For instance, circ-ZNF609 contains a 753-nucleotide open reading frame (ORF) that spans from the start codon to an in-frame stop codon. Notably, circ-ZNF609 has the potential to encode two distinct protein isoforms by both splicing-dependent and—independent mechanisms, marking the initial proof that endogenous circRNAs are capable of translation (48). Translationally competent circRNAs are emerging in muscle biology: while circZNF609 (IRES-driven) exemplifies a conserved myogenic paradigm in animals (31), chicken circFAM188B encodes the peptide circFAM188B-103aa that promotes skeletal-muscle satellite-cell proliferation, highlighting an endogenous coding output from avian circRNAs (62). The demonstration that circRNAs can be translated into functional peptides provides additional evidence supporting our hypothesis by showing that circRNAs serve as multifunctional regulatory molecules in poultry through both coding and non-coding mechanisms.

### 5.4 Transcriptional regulation

**Figure 2:**
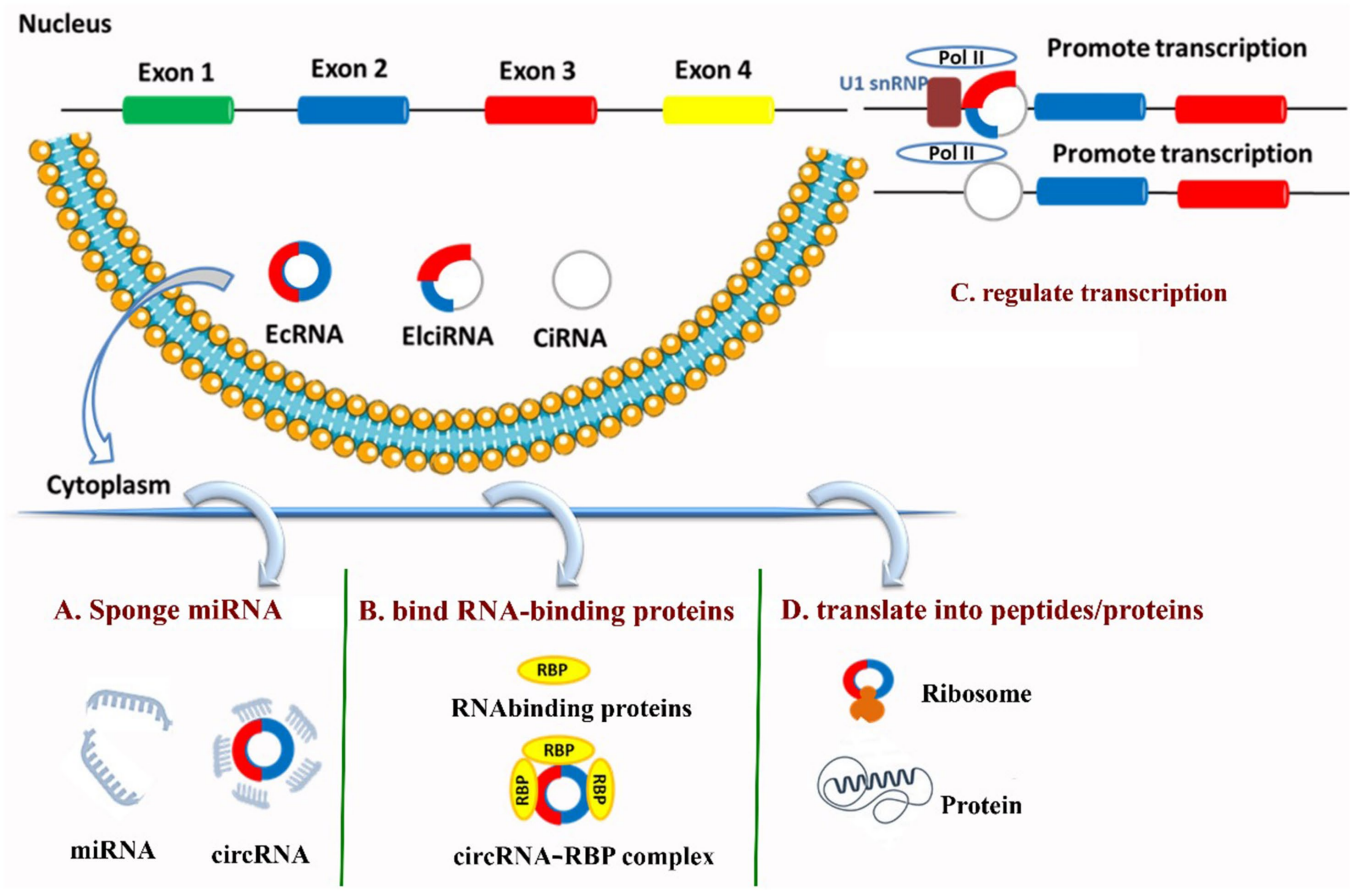
Four canonical circRNA functions [adapted from Zhang et al. (52)]. **(A)** Sponge miRNA and relieve repression on target mRNAs (ceRNA model). **(B)** Bind RNA-binding proteins and act as decoy/scaffold to modulate protein localization and activity. **(C)** Regulate transcription of parental genes, with EIciRNAs/ciRNAs engaging RNA polymerase II and/or U1 snRNP at promoters. **(D)** Translate into peptides/proteins via cap-independent initiation mediated by IRES elements or m6A.

Circular RNAs (circRNAs) are highly stable non-coding RNAs produced by back-splicing; beyond acting post-transcriptionally they also participate directly in transcriptional regulation and are themselves subject to complex transcriptional control. Some nuclear circRNAs, such as circular intronic RNAs (ciRNAs) and exon–intron circRNAs (EIciRNAs), interact with RNA polymerase II (Pol II), the enzyme that copies DNA into RNA, to boost the activity of the genes they come from. For example, ciRNAs such as ci-ANKRD52 enhance Pol II transcription at their host loci, while EIciRNAs (e.g., circEIF3J, circPAIP2) bind U1 snRNP and Pol II at promoters to stimulate transcription (35, 71, 72). Nuclear circRNAs can also recruit chromatin remodelers or transcription cofactors to promoters—chicken circRUNX2.2 recruits CHD9 to the RUNX2 promoter and promotes RUNX2 transcription—demonstrating a direct, locus-specific activating mechanism (27, 30). Figure 2 shows the four models of circRNA functions.

The circRNAs may also repress transcription through alternative mechanisms: some bind their parent loci to form R-loops (RNA–DNA hybrids) that cause Pol II pausing or termination (e.g., circSMARCA5), or they act as decoys/scaffolds that sequester transcription factors or RNA-binding proteins away from promoters, altering transcription factor binding and chromatin states (71, 73, 74). Epigenetic associations are common: circRNA loci frequently overlap regulatory regions marked by active histone marks (e.g., H3K27ac) and can be integrated into transcription factor networks (75, 76). Importantly, circRNAs can act both in cis (at their host gene) and in trans (on distant genes) by recruiting or sequestering regulatory proteins.

Conversely, circRNA biogenesis (their “transcriptional” control) is tightly regulated. Back-splicing competes with canonical linear splicing and is promoted by intron-pairing (complementary flanking sequences/inverted repeats), RNA-binding proteins (RBPs) that bridge splice sites (e.g., MBL, QKI), and lariat/exon-skipping pathways. In chickens, reverse complementary matches (RCMs), including elements overlapping CR1 repeats, facilitate exon circularization; deleting RCMs reduces circRNA formation (27, 30, 46). Biogenesis can thus reduce linear mRNA output (negative correlation between exonic circRNAs and parental mRNAs in some tissues) or, alternatively, correlate positively (as reported for some intronic circRNAs in chicken adipocytes), depending on context and mechanism. Post-transcriptional modifiers also affect circularization: ADAR1-mediated A → I editing can disrupt intronic base-pairing and inhibit circRNA formation (and has been implicated in AR-linked suppression of circRNAs in HCC), whereas m6A modification can promote production and translation of certain circRNAs with ORFs (48, 62, 77).

In sum, circRNAs both shape and are shaped by transcriptional programs: they modulate Pol II activity, recruit or sequester regulatory proteins, form R-loops, influence chromatin and TF binding, and compete with linear splicing—while their biogenesis is controlled by intronic architecture, RBPs, RNA editing, and epitranscriptomic marks. These two-way interactions make circRNAs integral players in transcriptional regulation and attractive candidates for functional studies and therapeutic targeting in poultry and other species (71, 75, 78, 79).

For poultry studies, beyond post-transcriptional miRNA sponging, avian circRNAs operate across multiple regulatory layers relevant to disease and production traits. A kinase/TF–focused ceRNA circuit is exemplified by 10:12574340|12607185, which interacts with gga-miR-92-5p and novel_miR_263 to influence STK10 (an AMPK-related kinase) and STAT5A during adipogenesis (33). At the transcriptional level, circRUNX2.2 elevates RUNX2 in tumorous spleens by acting in cis at the promoter and recruiting CHD9, illustrating that circRNAs can couple enhancer-like activity with ceRNA functions in Marek’s disease (27). Biogenesis competes with linear splicing, as Z:35565770|35568133 may alter processing of its parental gene ABHD17B in Gushi chicken adipocytes (33). Systems analyses map DE-circRNAs to lipid-metabolism hubs (CPT1A, AADAC, ACSS2, ACSL1, AGPAT2, FABP4, CYP26B1, FAAH), consistent with coordinated control of fatty-acid activation/transport and glycerolipid synthesis (33). Tissue-resolved ceRNA modules, novel_circ_PTPN2, novel_circ_CTNNA1, novel_circ_PTPRD, intersect with PPAR and fatty-acid pathways via specific miRNAs to regulate lipid genes across abdominal fat, back skin, and liver of *Gallus gallus* (64), while depot-specific candidates (circLCLAT1, circFNDC3AL, circCLEC19A, circARMH1) help partition intramuscular versus abdominal fat programs (65). Consistently, cross-tissue remodeling (41, 26, and 15 DE circRNAs in abdominal fat, back skin, and liver, respectively) converges on PPAR, glycerolipid, and fatty-acid metabolism, aligning circRNA regulation with carcass fatness phenotypes (64).

Collectively, these four regulatory mechanisms, miRNA sponging, protein scaffolding, translation, and transcriptional regulation, provide comprehensive evidence supporting our central hypothesis. The documented examples from poultry systems demonstrate that circRNAs indeed function as multifaceted key regulatory molecules that modulate disease pathogenesis and immune responses through diverse, interconnected mechanisms, establishing them as promising targets for diagnostic and therapeutic applications in avian health.

## 6 Role of circular RNA in diseases

The advent of high-throughput RNA sequencing and refined bioinformatics tools has revealed their widespread expression across viruses, plants, archaea, and animals, along with their pivotal roles in regulating cellular homeostasis and disease processes. Recent studies have confirmed the presence of circRNAs in both DNA and RNA viruses, where they play crucial roles such as evading the host’s immune response, contributing to disease pathogenesis, enhancing protein translation, serving as miRNA sponges, and regulating viral replication and cellular division. Endometrial fibrosis, endometriosis-related infertility, and pre-eclampsia (PE) are among the most common gynecological disorders worldwide, all of which have been linked to circRNAs (80). Most exemplars derive from mammalian research provides essential context for circRNA biology. These mammalian insights set the stage for understanding disease associations, while poultry-specific data and mechanisms are addressed separately in Section 7.

Recent data suggests that many circRNAs may interact with miRNAs, therefore participating in several physiological and pathological processes. For example, hsa_circ_0009361 modulates the expression of adenomatous polyposis coli 2 by interacting with miR-582, thus impeding the advancement of colorectal cancer (81). Microvascular complications are prevalent in diabetic retinopathy (DR) and may lead to blindness. Recent studies have correlated three RNA molecules—miRNAs, long noncoding RNAs (lncRNAs), and circRNAs—with the occurrence and development of diabetic retinopathy (DR), indicating their potential role as upstream regulators or participants in the functional processes related to this disease (82). Moreover, research indicates that circRNAs produced by viruses or differently expressed host circRNAs may function as potential biomarkers for viral infections (11). Along with their roles as supreme controllers of gene appearance, circRNAs present intriguing potential as novel biomarkers for cancer and other diseases (83).

Beyond these examples, the dysregulation of circRNAs is now recognized as a hallmark in a broad spectrum of human diseases, where they may act as either pathogenic drivers or protective modulators.

- Inflammatory Bowel Disease (IBD): Aberrant circRNA expression has been documented in ulcerative colitis and Crohn’s disease, affecting intestinal epithelial integrity, immune homeostasis, and fibrotic remodeling. For instance, circ_103516 correlates positively with TNF-*α* and IFN-γ levels while suppressing IL-10, thereby amplifying inflammation through miR-19b-1-5p sequestration. Other circRNAs such as circSMAD4 activate JAK2/STAT3 signaling, whereas circPABPN1 impairs autophagy by disrupting HuR–ATG16L1 interactions. Certain circRNAs also serve as non-invasive biomarkers capable of distinguishing Crohn’s disease from ulcerative colitis with high diagnostic accuracy (84).
- Neurological Disorders: The mammalian brain exhibits high circRNA abundance, with dynamic expression during development, aging, and neurodegeneration (85). For instance, circRNA dysregulation is strongly linked to Alzheimer’s disease (AD), with altered expression profiles correlating with cognitive decline. Key examples include: circAPP, which influences microglial polarization via the miR-1906/CLIC1 axis; CDR1as, which sponges miR-7, leading to α-synuclein buildup and impaired protein degradation; circHOMER1, downregulated in AD and potentially regulating PSEN1/PSEN2; circHDAC9, neuroprotective through miR-142-5p sponging; circLPAR1, promoting Aβ-induced apoptosis and inflammation; circNF1-419, enhancing autophagy via PI3K/Akt/AMPK/mTOR signaling; circAβ-a, generating Aβ polypeptides; circCORO1C, reducing miR-105 and increasing APP; circPSEN1, affecting TGF-β1/Notch pathways via miR-4668-5p and miR-5584-5p; and circRIMS2, whose m6A modification contributes to synaptic and memory deficits (63, 84, 86, 87).
- In Parkinson’s disease, circSLC8A1 is linked to oxidative stress via miR-128/miR-132; circ_0004381 and circ_0017204, early PD biomarkers; circ_0085869, circ_0004381, and circ_0090668, distinguishing disease stages; circEps15, which promotes dopaminergic recovery via miR-24/PINK1-PRKN mitophagy; circSV2b, restoring dopamine synthesis via miR-5107-5p/Foxk1/Akt1; and circPANK1 and circSNCA, which boost α-synuclein by sponging miR-7, driving neurotoxic aggregation. Comparable mechanistic roles are reported in amyotrophic lateral sclerosis, Huntington’s disease, schizophrenia, bipolar disorder, multiple sclerosis, and epilepsy (63, 84, 85).
- Cancer: circRNAs can function as oncogenes or tumor suppressors. In prostate cancer, circSMARCC1 promotes tumor-associated macrophage infiltration and M2 polarization, while in glioblastoma, circMTO1 and circHIPK3 facilitate tumor progression through miRNA sponging and pathway modulation. In colorectal cancer, circCCDC66 and circITGA7 regulate metastatic behavior, whereas in hepatocellular carcinoma, circATP5H enhances tumor growth via TNFAIP3 regulation through miR-138-5p (85, 88, 89).
- Autoimmune Diseases: Elevated CiRS-7 in rheumatoid arthritis disrupts mTOR signaling by sequestering miR-7. Multiple sclerosis patients display distinctive leukocyte circRNA profiles, highlighting potential biomarker applications. Similar immunoregulatory roles are observed in systemic lupus erythematosus and other immune-mediated disorders (63, 85, 86).
- Cardiovascular Diseases: circRNAs influence cardiomyocyte fate by modulating apoptosis, autophagy, pyroptosis, necroptosis, and ferroptosis through pathways such as NF-κB, PI3K/AKT, and TGF-β1. Examples include circANRIL in atherosclerosis and circDICAR in myocardial ischemia/reperfusion injury (84, 86).
- Pathogenic Infections: Both host- and pathogen-derived circRNAs participate in antiviral or proviral mechanisms. In viral infections, *hsa_circRNA_001387* and EBV-derived *circRPMS1* serve as nasopharyngeal carcinoma markers, while KSHV produces circRNAs from its PAN RNA region and HPV generates *circE7*, encoding the E7 oncoprotein linked to high-risk cancers. Coronaviruses, including SARS-CoV-2, produce circRNAs such as *circ_3205*, which promotes infection by sponging *miR-298* and upregulating PRKCE and KCNMB4. Due to their stability, long translation times, and low immunogenicity, circRNAs are promising candidates for durable viral and cancer vaccines (63, 84). In bacterial infections, *S. aureus* alters circRNA profiles in milk-derived extracellular vesicles, and reduced *circ_0009128* and *circ_0005836* in PBMCs may indicate tuberculosis (63).
- circRNAs hold strong potential as diagnostic and therapeutic tools due to their stability, disease- and tissue-specific expression, and presence in diverse biofluids such as blood, saliva, urine, cerebrospinal fluid, and exosomes. They can aid in diagnosis, prognosis, and treatment monitoring. Therapeutic strategies under investigation include RNA interference targeting back-splice junctions, viral vector-mediated overexpression, nanocarrier or exosome-based delivery, and synthetic circRNAs or aptamers as direct therapeutic agents (63, 85, 88, 89).

This mechanistic framework, immune hijacking and metabolic reprogramming, provides the conceptual foundation for interpreting circRNA functions in poultry diseases, where similar regulatory principles likely operate through species-specific molecular networks.

## 7 Research progress of circular RNA in poultry

**Figure 3:**
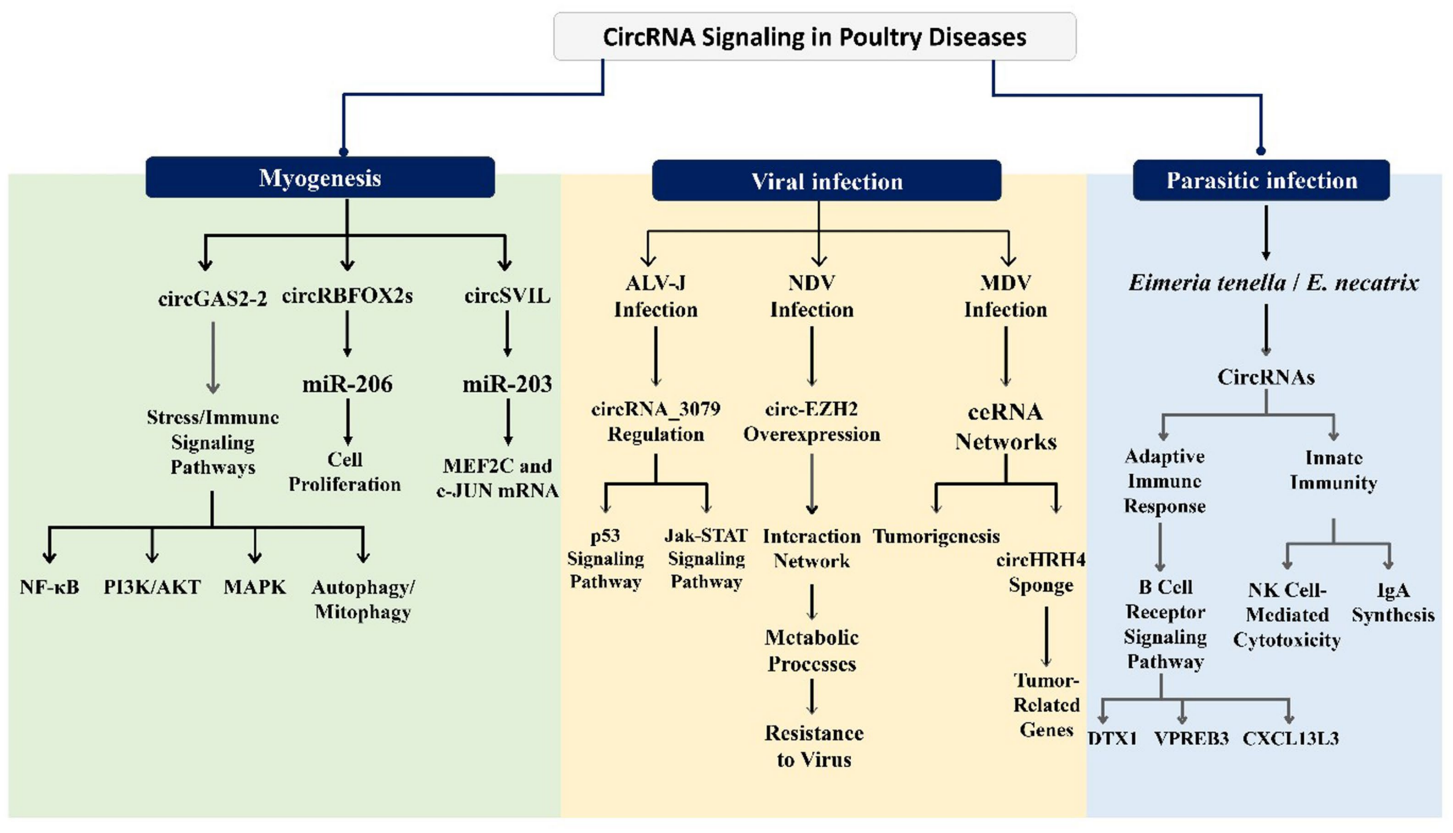
Mechanistic map of circRNA-centered signaling across major poultry diseases.

This section presents direct experimental evidence from poultry systems or avian cell/tissue datasets that test our central hypothesis, and developmental processes provide species-specific validation of their function as key regulatory molecules in avian disease pathogenesis and immune responses. Recent investigations have begun to illuminate the specific circRNAs associated with poultry diseases such as infectious bronchitis, avian influenza, and coccidiosis. Analyzing the expression patterns, functional roles, and diagnostic potential of circRNAs in these contexts represents a crucial step toward harnessing their therapeutic and diagnostic capabilities in poultry health. circRNAs have a vital function in various biological processes and the progression of disorders. For instance, the investigation of circRNA_3079 in chickens reveals that it is a stable circular transcript predominantly located in the cytoplasm (90). This circRNA is broadly expressed across different tissues, particularly in the lung, spleen, lymph nodes, and bursa of Fabricius. circRNA_3079 may indirectly regulate the process of avian leukosis virus subgroup J (ALV-J) infection. Bioinformatics analyses have indicated that circRNA_3079 and its predicted target genes are enriched in several mechanisms that are associated with immunity and tumors, such as the p53 signaling pathway and the Jak–STAT signaling pathway (57). Additionally, circRNAs have been shown to act as miRNA sponges that regulate chicken muscle development. For instance, Chen et al. verified that circFGFR2 promotes myoblast differentiation and proliferation by interacting with miR-133a-5p and miR-29b-1-5p (30). Thus, circRNAs significantly impact various tissues and organs in poultry, playing an essential role in health and disease. Figure 3 provides a system-level snapshot of how poultry circRNAs wire ceRNA networks into core signaling pathways to shape outcomes across viral, parasitic, and non-infectious conditions.

This figure highlights circRNA functions in myogenesis, viral infections, and parasitic infections. In muscle development, circRNAs such as circGAS2-2, circRBFOX2s, and circSVIL regulate cell proliferation, immune/stress pathways (NF-κB, PI3K/AKT, MAPK, autophagy), and transcription factors (MEF2C, c-JUN). During viral infections, circRNA_3079 modulates the p53 and Jak–STAT pathways in ALV-J, circ-EZH2 enhances resistance to NDV via metabolic regulation, and a circHRH4 ceRNA network drives tumorigenesis in MDV. In parasitic infections (*Eimeria tenella* and *E. necatrix*), circRNAs influence adaptive immunity (B cell receptor signaling via DTX1, VPREB3) and innate immunity (NK cell-mediated cytotoxicity, IgA synthesis, CXCL13L3).

### 7.1 Viral infection

#### 7.1.1 Avian leukosis virus subgroup J (ALV-J)

The oncogenic exogenous retrovirus known as Avian Leukosis Virus Subgroup J (ALV-J) leads to many tumors, slowed growth, high mortality rates, and decreased immunity, and it has been related to poor egg-laying abilities. ALV-J infection fundamentally rewires the host circRNA landscape, creating a molecular environment that promotes tumorigenesis through coordinated disruption of immune surveillance mechanisms. The evidence for this regulatory hijacking comes from multiple converging studies. Zhang et al. used high-throughput transcriptome sequencing of ALV-J–infected chicken macrophages and fibroblasts to identify 7,684 differentially expressed circRNAs. RT-qPCR validation confirmed their association with immune regulation and oncogenic signaling, suggesting roles in ALV-J pathogenesis (28). Critically, these changes were not random but showed systematic enrichment in immune regulatory and oncogenic signaling pathways, suggesting that circRNA perturbation is a central mechanism by which ALV-J establishes and maintains tumorigenic conditions.

The magnitude of this circRNA response indicates that viral oncogenesis depends on comprehensive reprogramming of the host regulatory network rather than simple immune evasion. Supporting this concept, circRNA_3079 and circRNA_3238 emerge as key regulatory nodes during ALV-J infection, with bioinformatics analyses revealing their integration into p53 and JAK/STAT signaling cascades, pathways fundamental to both immune defense and tumor suppression (57). This coordinated targeting of multiple tumor suppressor pathways through circRNA modulation provides a mechanistic explanation for ALV-J’s potent oncogenic effects and suggests that circRNA-mediated regulation represents a vulnerability that could be therapeutically exploited.

As one of the major non-coding RNAs, circRNAs have essential functions in different biological processes, consisting of skeletal muscle development (91). They uniquely regulate both muscle growth in humans and animals and associated physiological and pathological aspects (92). However, research on circRNAs regulating skeletal muscle development in chickens remains limited, particularly during embryonic stages. The 14-day embryo represents a critical period for myoblast proliferation and differentiation into myotubes, which ultimately fuse into muscle fibers by 20 days of age. Wu et al. (91) collected leg muscles from 14 to 20-day-old Bian chicken embryos for RNA-seq, aiming to recognize key circRNAs involved in expansion of skeletal muscle.

These findings from ALV-J studies provide direct evidence supporting our hypothesis, demonstrating that circRNAs function as key regulatory molecules in poultry viral pathogenesis through their differential expression patterns, association with immune and oncogenic pathways, and potential roles in disease resistance mechanisms.

#### 7.1.2 Newcastle disease virus (NDV)

Newcastle disease virus (NDV) is avian paramyxovirus serotype-1 (APMV-1), which was first isolated from pigeons in the Middle East in 1978 (93). NDV infection demonstrates that host circRNAs can function as molecular switches that determine viral replication outcomes through metabolic reprogramming. This regulatory model is supported by compelling functional evidence from Chen et al., who identified 86 differentially expressed circRNAs during NDV infection, with pathway enrichment analyses revealing systematic targeting of host metabolic processes (25). The key insight emerges from circ-EZH2, which exemplifies how individual circRNAs can serve as master regulators of antiviral resistance. Functional validation proves that circ-EZH2 acts as a critical determinant of infection outcome: its overexpression significantly inhibited velogenic NDV replication, while knockdown promoted viral propagation (25). This bidirectional effect establishes circ-EZH2 as a host restriction factor that controls viral replication through downstream metabolic regulation. The mechanistic significance extends beyond individual circRNAs—the coordinated expression changes in 86 circRNAs suggest that NDV resistance depends on comprehensive metabolic reprogramming mediated by multiple circRNA-mRNA-miRNA regulatory networks. This finding reframes NDV pathogenesis from simple viral cytotoxicity to a battle for control over host cellular metabolism, with circRNAs serving as the molecular battlefield where infection outcomes are determined. The NDV infection studies strongly support our hypothesis by showing that circRNAs, particularly circ-EZH2, function as key regulatory molecules in poultry antiviral responses through their ability to modulate metabolic processes and directly inhibit viral replication, demonstrating their therapeutic potential.

#### 7.1.3 Marek’s disease virus (MDV)

The Marek’s disease virus (MDV), which causes Marek’s disease, poses a significant risk to the poultry sector as it is a lymphotropic neoplastic disease. RNA sequencing of tumorous spleens (TS) was completed in order to investigate the expression profiles of host circRNAs and microRNAs (miRNAs). Two spleen types were analyzed: spleens from survivors (SS) without lesions after MDV infection and non-infected chicken spleens (NS). This study identified a total of 2,169 circRNAs, with more than 80% derived from exons. Among these, 113 circRNAs were classified as differentially expressed circRNAs (DECs) (56). There have been more and more circRNAs discovered in recent years in hepatocellular carcinoma (HCC), assisting in the development of tumors and their spread, and showing possible use as biomarkers by their roles as competitive endogenous RNAs (ceRNAs), acting as miRNA sponges, possibly by interactions with proteins that bind RNA (RBPs). Chasseur conducted full-length sequencing and viral circRNA expression analysis in MDV infections and discovered that circRNAs from the same essential virulence genes were highly expressed *in vivo* (94).

Viral circRNA sequences described from *in vitro* and *in vivo* infections showed many noncanonical junctions, which is surprising because the detected back-splice junctions do not follow a unique canonical pattern consistent with the U2-dependent splicing machinery. Qiu conducted a study on when the avian leukosis virus, subgroup J, causes cancer in hens, and competing endogenous RNA networks are shown by circular RNA and mRNA profiling. Transcript structure analysis revealed that chicken circRNAs had similar GC content and comparatively shorter transcripts than mRNAs and lncRNAs. Differential expression analysis identified 152 circRNAs, including 106 up-regulated and 46 down-regulated circRNAs (12). Through the comparison of differentially expressed circRNA host genes and mRNAs, alongside ceRNA network analysis, many tumor or immune-related genes were found, including four genes (Dock4, Fmr1, Zfhx3, Ralb) and two genes (Mll, Aoc3) associated with the ceRNA network. They also identified a circRNA generated from the HRH4 gene inside the ceRNA network, termed circHRH4 (95–98). These MDV investigations provide compelling evidence for our hypothesis, revealing that circRNAs serve as key regulatory molecules in poultry oncogenic viral diseases through multiple mechanisms including transcriptional regulation (circRUNX2.2), ceRNA networks (circHRH4), and both host and viral circRNA interactions that modulate tumorigenesis.

### 7.2 Parasitic and bacterial infections (coccidiosis; *Eimeria tenella*/*E. necatrix*)

circRNAs are long non-coding RNAs that perform vital functions in inflammatory reactions and a number of infectious illnesses. These circRNAs are not merely bystanders but are actively involved in shaping the host’s immune response to the parasite. The *E. tenella* parasitizes mostly resulting in bloody stools and bleeding of the cecum epithelium in chickens (99). High-throughput sequencing was used to detect circRNAs in the cecal tissues of chickens from the susceptible (JS), resistant (JR), and control (JC) groups on day 4.5 post-infection. This research found 104 differentially expressed circRNAs associated with pathways relevant to *Eimeria tenella* infection (26). In poultry, Chen et al. (100) found that chicken ammonia poisoning elevated the expression of five differentially expressed circRNAs. Liu et al. (9) discovered that 27 differently expressed circRNAs contributed to the immune response to chronic bursal infection in chickens. Fan et al. (101) performed transcriptome sequencing on the small intestine tissues of chickens infected with *E. necatrix* and identified 13 differentially expressed circRNAs, including circRNA2673, circRNA3106, and circRNA1579, which were essential to the *E. necatrix* infection process. During *E. tenella* infection, circRNAs function as molecular selectors that shape resistance or susceptibility by directing innate versus adaptive immune pathways. Resistant birds showed circRNA enrichment in NF-κB and NK cell cytotoxicity pathways, while susceptible birds displayed adaptive pathway bias. Key circRNA–gene pairs (e.g., circRNA2202/*DTX1*, circRNA4338/*VPREB3*, *CXCL13L3*) illustrate their role in fine-tuning immune cell fate and determining infection outcomes (26). The functional enrichment of these differentially expressed circRNAs was significantly prevalent in the adaptive immune response and B cell receptor signaling pathways (101).

Adaptive immunity regulates antigen-specific immune responses to prevent the pathogen from colonizing and proliferating inside the host. It is an essential element of anti-coccidial protective immunity (102). The B cells are essential components of adaptive immune responses in avians (103). B cells with identical surface immunoglobulin may persist in division and proliferation to enhance host immunity after coccidia infection, during which they produce antigen-specific surface immunoglobulin molecules that bind to the antigen (104). Differentially expressed circRNAs were considerably enriched in the natural killer cell-mediated cytotoxicity pathway, associated with the innate immunity to coccidian infection and involved in the defense against coccidia’s invasion of the gut mucosa (105).

Therefore, in susceptible chickens, DE circRNAs were associated with the adaptive immune response. In contrast, in resistant birds, the enriched pathways included NF-kappa B signaling and natural killer (NK) cell-mediated cytotoxicity such as IgA synthesis during coccidia infection. Specific circRNAs, such as *circRNA2202* and *circRNA0759*, were found to be associated with the *DTX1* gene, while *circRNA4338* was linked to *VPREB3* and *CXCL13L3*, all of which are involved in immune regulation (26). This highlights how circRNA-mediated ceRNA networks can differentiate between resistant and susceptible host responses, providing valuable insights into the molecular mechanisms of disease resistance in poultry. These results indicated that these circRNAs have a role in coccidian infection by affecting chickens’ innate and adaptive immune responses. Together, these coccidiosis studies provide strong evidence supporting our hypothesis by demonstrating that circRNAs function as key regulatory molecules in poultry parasitic diseases through their modulation of both adaptive and innate immune responses, with specific circRNAs serving as molecular switches that differentiate between resistant and susceptible host responses.

### 7.3 Muscle development processes (myogenesis)

Myogenesis in poultry operates through temporally coordinated waves of circRNA expression that synchronize metabolic reprogramming with cell fate transitions, ensuring that muscle fiber formation proceeds in precise developmental sequence. Studies have identified a plethora of circRNAs that are dynamically expressed during chicken embryonic and post-hatching muscle development. These myogenesis-associated circRNAs form intricate ceRNA networks that regulate genes involved in muscle metabolism, including glycolysis/gluconeogenesis, amino acid biosynthesis, and energy production (59). For example, a circRNA-miRNA-mRNA network was constructed involving 68 circRNAs, 361 miRNAs, and 599 mRNAs, all showing a tendency to be upregulated with age, consistent with the rapid muscle growth observed after hatching (59). These circRNAs are not only involved in the intrinsic regulation of muscle development but also exhibit crosstalk with stress and immune signaling pathways. This suggests that the physiological state of the bird, including stress levels, can influence muscle growth through circRNA-mediated mechanisms, integrating immune, metabolic, and developmental processes (106). This integration ensures that muscle growth proceeds only when metabolic resources are sufficient and immune challenges are minimal, optimizing developmental efficiency in response to environmental conditions.

To investigate circRNA expression during duck muscle development, Liu et al. (107) collected pectoral muscle samples from Shan Ma ducks at two critical embryonic time points: day 13 (E13), when myoblasts remain undifferentiated, and day 19 (E19), when myoblasts have differentiated. RNA sequencing identified 16,622 circRNAs, of which nearly 80% were exonic circular RNAs; importantly, 260 exhibited differential expression between E19 and E13. The functional evidence demonstrates that specific circRNAs serve as molecular gatekeepers controlling developmental transitions. CircGAS2-2 exemplifies this gatekeeper function: its overexpression accelerated cell cycle progression and enhanced myoblast proliferative capacity, while knockdown blocked cell cycle advancement and reduced proliferative viability (107). This bidirectional control establishes circGAS2-2 as a master regulator that determines when myoblasts transition from proliferation to differentiation phases. The total quantity of muscle fibers is the critical determinant of muscle mass, controlled throughout embryogenesis or early post-hatch in chickens (108). Likewise, the circRBFOX2s was confirmed to promote cell proliferation via their association with miR-206 (109).

RNA sequencing was conducted on the leg muscles of female Xinghua (XH) chickens at three developmental stages: 11 embryonic days (E11), 16 embryonic days (E16), and 1-day post-hatch (P1) to identify circRNAs involved in chicken embryonic skeletal muscle development. A total of 13,377 circRNAs were discovered, with 3,036 exhibiting high expression levels, mainly originating from coding exons. Similarly, 462 circRNAs exhibited differential expression (fold change > 2; *p*-value < 0.05), with their parental genes associated with biological activities in muscle tissue. Among them, 946 exonic circRNAs were identified to possess one or more miRNA-binding sites for 150 known miRNAs. Ouyang examined the impact of circSVIL on skeletal muscle development and identified the levels of circSVIL expression in the leg muscle of embryos from E10 to P1 (110). The expression level of circSVIL was elevated during the late embryonic development of skeletal muscle. The dual-luciferase test, RNA immunoprecipitation, and biotin-coupled miRNA pull-down revealed that chicken circSVIL acts as a sponge for miR-203, therefore enhancing the mRNA levels of MEF2C and c-JUN (111). These mechanistic studies collectively demonstrate that circRNAs function as critical regulatory molecules during myogenesis through their ability to modulate miRNA availability and subsequently influence target gene expression. The consistent observation of circRNA-miRNA-mRNA regulatory networks across different developmental stages and muscle types provides strong evidence supporting our hypothesis that circRNAs serve as key mediators of normal physiological processes in poultry, extending their regulatory influence beyond disease contexts to fundamental developmental biology, establishing them as promising diagnostic biomarkers and therapeutic targets.

## 8 Challenges in circRNA research and therapeutic translation

Circular RNAs (circRNAs) have emerged as an exciting frontier in RNA biology, expanding beyond the earlier discoveries of microRNAs to reveal critical roles in gene regulation and disease processes (6, 112, 113). Their covalently closed-loop structure grants high stability and resistance to exonucleases, while their diverse and often tissue-specific expression patterns make them promising candidates for diagnostic and therapeutic applications. In poultry, circRNAs have been linked to immune defense, metabolism, growth, and disease susceptibility, offering opportunities for novel interventions. However, their translation from bench to field faces multiple interconnected scientific and technical barriers that must be addressed systematically before circRNA-based strategies can be reliably implemented in agricultural or clinical settings.

A central research challenge is accurate identification and quantification of circRNAs in avian species. The closed-loop structure complicates detection using conventional RNA sequencing pipelines, which are optimized for polyadenylated transcripts (17, 114). Even gold-standard enrichment workflows, combining rRNA depletion, RNase R digestion, and divergent primer-based RT-qPCR, introduce biases that can skew abundance estimates. Bioinformatics tools such as CIRI-quant and Sailfish-cir improve detection of back-splice junctions but remain prone to errors in low-abundance or alternatively spliced transcripts, especially in complex poultry tissues (115). The lack of standardized protocols, spike-in controls, and universally accepted nomenclature further hampers reproducibility and biomarker validation.

Beyond detection, functional elucidation of circRNAs remains limited. Although high-throughput sequencing has revealed thousands of circRNAs across tissues such as skeletal muscle, liver, spleen, and granulosa cells (7), few have undergone rigorous functional validation in poultry. For example, CRISPR-mediated knockout of circHIPK3 in broilers reduced abdominal fat but impaired antiviral antibody responses, illustrating the delicate balance between production traits and immune fitness (116, 117). Moreover, environmental stressors such as diet composition and heat stress dynamically alter circRNA expression, effects that are difficult to replicate *in vitro* due to the scarcity of physiologically relevant avian cell models.

One of the key obstacles in targeted circRNA modulation is achieving tissue- and cell-type specificity. While some circRNAs exhibit highly restricted expression patterns, others are broadly distributed, making them less suitable as selective biomarkers or therapeutic targets (118). Current molecular strategies, such as RNA interference, antisense oligonucleotides (ASOs), and expression plasmids, often lack the precision required to prevent off-target effects, and nanoparticle-based delivery platforms remain in developmental stages (119). Effective delivery remains a central challenge: viral vectors provide stable transduction but are constrained by packaging limits and potential immunogenicity, whereas non-viral systems like lipid nanoparticles face low encapsulation efficiency, rapid clearance, and inefficient endosomal escape (6, 120). In complex organisms such as poultry, delivering therapeutic agents, including small molecules or ASOs, without provoking immune reactions or causing tissue damage is particularly difficult. These limitations hinder treatment efficacy, especially for systemic diseases like viral infections or metabolic disorders. Continued progress in engineering viral vectors and next-generation nanoparticle systems with enhanced precision and tissue selectivity may help overcome these barriers (121).

Another emerging barrier is synthetic circRNA immunogenicity (122). While engineered circRNAs offer potential for therapeutics, vaccines, and protein expression due to their stability, they can still activate innate immune receptors such as RIG-I, MDA5, and PKR if they contain immunostimulatory motifs or contaminants (120). The absence of the m6A alteration was proposed to differentiate foreign circRNAs from endogenous circRNAs (123). Current research is looking for ways to lessen the immunogenicity of synthetic circRNA, such as wrapping them in RBPs and applying chemical changes. Strategies to mitigate immune activation—including chemical modifications and immunologically inert delivery vehicles—are promising but not yet foolproof, especially in large-animal models.

Finally, biogenesis-related complications such as mis-splicing remain a persistent issue. Overexpression systems relying on inverted repeats or intronic complementary sequences can generate unintended linear or aberrantly spliced products, interfering with functional readouts and potentially sequestering regulatory molecules (6, 112). While long-read sequencing can improve structural characterization, it remains labor-intensive and is not yet compatible with high-throughput production.

Collectively, these challenges, spanning detection, functional validation, targeting specificity, delivery efficiency, immunogenicity, and biosynthetic fidelity, underscore the need for a cohesive research roadmap. Addressing these interconnected issues in a staged manner is essential. The most immediate priority should be establishing standardized detection and quantification pipelines for poultry circRNAs, as reliable identification underpins all downstream functional and translational work. Once robust profiling platforms are in place, targeted modulation strategies and delivery systems can be developed and optimized with greater precision, paving the way for circRNA-based diagnostics and therapeutics that can meaningfully enhance poultry health and productivity. Addressing these interconnected challenges through coordinated research efforts will be essential for translating the strong evidence supporting our hypothesis, that circRNAs serve as key regulatory molecules in poultry diseases, into practical diagnostic tools and therapeutic interventions that can meaningfully enhance poultry health and productivity.

## 9 Conclusions and future perspectives

circRNAs are now recognized as important regulators in cellular physiology and disease, including antiviral immune responses, yet our understanding in poultry remains fragmentary. A persistent challenge is the absence of a unified nomenclature system—current naming conventions based on host genes (e.g., circMTO1) or functions (e.g., ciRS-7, MFACR) hinder cross-study comparisons and database integration. Equally critical are technical barriers in accurate detection, as most standard RNA sequencing pipelines are not optimized for circular transcripts, leading to inconsistent quantification. Functionally, while circRNAs have been linked to modulation of innate immunity and protection against viral infections, the majority of identified circRNAs still lack experimental validation, limiting their translational potential. To address these gaps, we propose four priority actions: (1) develop standardized, poultry-adapted pipelines for circRNA detection and quantification, including reference spike-ins and shared bioinformatic criteria; (2) establish a consensus nomenclature endorsed by the avian research community to facilitate data sharing; (3) invest in targeted functional assays—such as CRISPR/Cas13-mediated knockdowns—in physiologically relevant avian cell models to link circRNAs to immune phenotypes; and (4) accelerate delivery technology development, particularly nanoparticle and exosome systems, for precise circRNA modulation *in vivo*. By systematically tackling these limitations through coordinated methodological, functional, and translational research, circRNA-based diagnostics and therapeutics could transition from exploratory discovery to practical tools for enhancing poultry health, productivity, and disease resilience.
